# Supplementary material for: Factors Associated with Vitamin D Testing: A Population-Based Cohort Study in Queensland, Australia
Source: Nutrients. 2025 Aug 4;17(15):2549. doi: 10.3390/nu17152549 (PMC12348133; doi:10.3390/nu17152549)
Supplement: Supplementary file 1 [file nutrients-17-02549-s001.zip › nutrients-3735692-supplementary.pdf]

Supplementary Methods S1. Explanatory variables

This section describes the ascertainment of all explanatory variables, relevant outcome(s) of interest, and how they were treated in our regression models. We used data from the baseline survey, Medicare Benefits Schedule (MBS), Pharmaceutical Benefits Scheme (PBS), Queensland Cancer Register (QCR), and pathology records to ascertain explanatory variables. MBS and PBS data were available up to 30 June 2020. QCR data were available up to 31 December 2021.

Table SA includes relevant information on the explanatory variables. Table SB lists all Anatomical Therapeutic Chemical (ATC) code used to calculate Rx-Risk score. Table SC shows the coefficients of the logistic regression model for the association between skin phenotypes and skin cancer outcome, from which we derived the weights used to construct a skin phenotype score.

Table SA. List of explanatory variables, corresponding outcome(s), and how they were treated in regression models

Explanatory variable	Source(s) of information	Outcome(s)[1]	Fixed / time-varying[2]
Sex	Baseline survey	First vitamin D test / Repeat vitamin D tests / Vitamin D deficiency in the first test	Fixed
Age at cohort entry (2011, 2015)	Age = 2011 (or 2015) – year of birth (from baseline survey)	First vitamin D test / Vitamin D deficiency in the first test	Fixed
Age at time of prior vitamin D test	Age = year of prior test – year of birth	Repeat vitamin D tests	Time-varying
Ancestry origin	Baseline survey	First vitamin D test / Vitamin D deficiency in the first test	Fixed
Skin phenotype	Based on skin color, propensity to burning, skin tanning, natural hair color (from baseline survey) (further details are provided in Table SC)	First vitamin D test / Vitamin D deficiency in the first test	Fixed
Socioeconomic Indexes for Areas (SEIFA) category	Baseline postcode and Australian Bureau of Statistics data	First vitamin D test / Vitamin D deficiency in the first test	Fixed
BMI at baseline	Baseline survey	First vitamin D test / Vitamin D deficiency in the first test	Fixed
History of regular smoking at baseline	Baseline survey	First vitamin D test / Vitamin D deficiency in the first test	Fixed
Alcoholic drinks per week at baseline	Baseline survey	First vitamin D test / Vitamin D deficiency in the first test	Fixed

<table>
<tr><th>Explanatory variable</th><th>Source(s) of information</th><th>Outcome(s)[1]</th><th>Fixed / time-varying[2]</th></tr>
<tr><td>Sun exposure in the year prior to baseline</td><td>Information on hours spent outdoors in the sun on weekdays and weekends from baseline survey was combined. For each question, we chose the midpoint of the range as the amount per day as per the following table.
<table>
<tr><th>Time per day</th><th>Hours</th></tr>
<tr><td>0-1 hour</td><td>0.5</td></tr>
<tr><td>1-2 hours</td><td>1.5</td></tr>
<tr><td>2-4 hours</td><td>3.0</td></tr>
<tr><td>≥4 hours</td><td>6.0</td></tr>
</table>
• Outdoors hours on weekdays = 5 x hours per day
• Outdoors hours on weekends = 2 x hours per day
• Total hours per week = hours on weekdays + hours on weekends.
Total hours per week was then categorized into four groups:
• Low: ≤3.5 hours/week;
• Medium: >3.5 to ≤10 hours/week;
• High: >10 to ≤25 hours/week; and
• Very high: >25 hours/week</td><td>First vitamin D test / Vitamin D deficiency in the first test</td><td>Fixed</td></tr>
<tr><td>Sunscreen use in the year prior to baseline</td><td>Baseline survey</td><td>First vitamin D test / Vitamin D deficiency in the first test</td><td>Fixed</td></tr>
<tr><td>GP visits in 12 months before cohort entry</td><td># MBS claims (for GP visits) in 12 months before the cohort entry date.
<u>MBS items:</u> 3-51, 195, 197, 199, 597, 599, 2497-2559, 5000-5067</td><td>First vitamin D test</td><td>Fixed</td></tr>
<tr><td>GP visits per year before the preceding test</td><td># MBS claims (for GP visits) between the previous 2 tests or 12 months before the first test, then being standardized to one year (by dividing for duration between tests).
<u>MBS items</u>: same as in GP visits in 12 months before cohort entry.</td><td>Repeat vitamin D tests</td><td>Time-varying</td></tr>
<tr><td>Rx-Risk comorbidity index at cohort entry</td><td>ATC code from PBS claims in 12 months before the cohort entry date.
<u>ATC code</u>: Refer to Table SB (Supplementary methods 1).</td><td>First vitamin D test</td><td>Fixed</td></tr>
<tr><td>Rx-Risk comorbidity index at time of test</td><td>ATC code from PBS claims between the previous 2 tests or 12 months before the first test.
<u>ATC code</u>: Refer to Table SB (Supplementary methods 1).</td><td>Repeat vitamin D tests</td><td>Time-varying</td></tr>
<tr><td>Result of previous vitamin D test</td><td>Pathology data</td><td>Repeat vitamin D tests</td><td>Time-varying</td></tr>
</table>

Explanatory variable	Source(s) of information	Outcome(s)[1]	Fixed / time-varying[2]
Skin cancers excised prior to baseline	Baseline survey	First vitamin D test / Vitamin D deficiency in the first test	Fixed
Skin cancers treated with cryotherapy prior to baseline	Baseline survey	First vitamin D test / Vitamin D deficiency in the first test	Fixed
Keratinocyte cancer subsequent to baseline	Date of first keratinocyte cancer treatment claim from MBS<br>MBS items: 31255, 31260, 31265, 31270, 31275, 31280, 31285, 31290 (between 2011 and 2015); 31356, 31358, 31359, 31361, 31363, 31365, 31367, 31369 (between 2016 and 2019).	First vitamin D test	Time-varying
Keratinocyte cancer subsequent to baseline	Date of first keratinocyte cancer treatment claim from MBS was compared to see if it preceded the first test (Yes/No).	Vitamin D deficiency in the first test	Fixed
Invasive melanoma subsequent to baseline	Date of first invasive melanoma diagnosed from QCR	First vitamin D test	Time-varying
Invasive melanoma subsequent to baseline	Date of first invasive melanoma diagnosed from QCR was compared to see if it preceded the first test (Yes/No)	Vitamin D deficiency in the first test	Fixed
In situ melanoma subsequent to baseline	Date of first in situ melanoma diagnosed from QCR	First vitamin D test	Time-varying
In situ melanoma subsequent to baseline	Date of first in situ melanoma was compared to see if it preceded the first test (Yes/No)	Vitamin D deficiency in the first test	Fixed
Ever been prescribed with osteoporosis medication	From PBS: Date of first prescription<br>ATC code: all M05 items.	First vitamin D test	Time-varying
Ever been prescribed with osteoporosis medication	Date of first prescription was compared to see if it preceded the first test (Yes/No).<br>ATC code: all M05 items.	Vitamin D deficiency in the first test	Fixed
Recent osteoporosis medication	Interval of 90 days from the first prescription.<br>ATC code: all M05 items.	First vitamin D test	Time-varying
Ever been prescribed with antiepileptic medication	From PBS: Date of first prescription.<br>ATC code: All N03 items.	First vitamin D test	Time-varying
Ever been prescribed with antiepileptic medication	Date of first prescription was compared to see if it preceded the first test (Yes/No).<br>ATC code: All N03 items.	Vitamin D deficiency in the first test	Fixed
Recent antiepileptic medication	Interval of 90 days from the first prescription.<br>ATC code: All N03 items.	First vitamin D test	Time-varying

Explanatory variable	Source(s) of information	Outcome(s)[1]	Fixed / time-varying[2]
Ever been prescribed with MHT	From PBS: Date of first prescription.<br>PBS items: 10146B, 8244X, 8425K, 8426L, 10142T, 8427M, 8428N, 1663M, 8485N, 8761D, 8762E, 8311K, 8274L, 1664N, 8125P, 8763F, 8140K, 8286D, 8486P, 8764G, 8765H, 8126Q, 8312L, 1781R, 1771F, 10203B, 2323G, 2993M, 2321E	First vitamin D test	Time-varying
Ever been prescribed with MHT	Date of first prescription was compared to see if it preceded the first test (Yes/No).<br>PBS items: Same as in ***Ever been prescribed with MHT***.	Vitamin D deficiency in the first test	Fixed
Recent MHT	Interval of 90 days from the first prescription.<br>PBS items: Same as in ***Ever been prescribed with MHT***.	First vitamin D test	Time-varying

ATC = Anatomical Therapeutic Chemical, BMI = Body mass index, GP = General Practitioner, MHT = Menopausal hormone therapy, MBS = Medicare Benefits Schedule, PBS = Pharmaceutical Benefits Scheme, QCR = Queensland Cancer Register.

[1]The outcome(s) of which the associations with the explanatory variable were assessed; [2]How the explanatory variable was treated in the regression model for the outcome.

Table SB. Rx-risk comorbidity category and corresponding Anatomical therapeutic chemical (ATC) code[1]

No.	Category	ATC code
1.	Alcohol dependency	N07BB01–N07BB99
2.	Allergies	R01AC01–R01AD60, R06AD02–R06AX27, R06AB04
3.	Anticoagulants	B01AA03–B01AB06, B01AE07, B01AF01, B01AF02, B01AX05
4.	Antiplatelets	B01AC04–B01AC30
5.	Anxiety	N05BA01–N05BA12, N05BE01
6.	Arrhythmia	C01AA05, C01BA01–C01BD01, C07AA07
7.	Benign prostatic hyperplasia	G04CA01–G04CA99, G04CB01, G04CB02*
8.	Bipolar disorder	N05AN01
9.	Chronic airway disease	R03AC02–R03DC03, R03DX05
10.	Congestive heart failure	C03DA02–C03DA99, C07AB02—if PBS item code is 8732N, 8733P, 8734Q, 8735R, C07AB07, C07AG02, C07AB12, C03DA04 (C03CA01– C03CC01 and C09AA01–C09AX99, C09CA01– C09CX99)†
11.	Dementia	N06DA02–N06DA04, N06DX01
12.	Depression	N06AA01–N06AG02, N06AX03–N06AX11, N06AX13–N06AX18, N06AX21–N06AX26
13.	Diabetes	A10AA01–A10BX99
14.	Epilepsy	N03AA01–N03AX99
15.	Glaucoma	S01EA01–S01EB03, S01EC03–S01EX99
16.	Gastrooesophageal reflux disease	A02BA01–A02BX05
17.	Gout	M04AA01–M04AC01
18.	Hepatitis B	J05AF08, J05AF10, J05AF11
19.	Hepatitis C	J05AB54, L03AB10, L03AB11, L03AB60, L03AB61, J05AE14, J05AE11–J05AE12, J05AX14, J05AX15, J05AX65, J05AB04
20.	HIV	J05AE01–J05AE10, J05AF12–J05AG05, J05AR01–J05AR99, J05AX07–J05AX09, J05AX12, J05AF01–J05AF07, J05AF09

No.	Category	ATC code
21.	Hyperkalaemia	V03AE01
22.	Hyperlipidaemia	A10BH03‡, C10AA01–C10BX09
23.	Hypertension	C03AA01–C03BA11, C03DB01, C03DB99, C03EA01, C09BA02–C09BA09, C09DA02–C09DA08, C02AB01–C02AC05, C02DB02–C02DB99 (C03CA01–C03CCO1 or C09AA01- C09A×99 or C09CA01–C09CX99)§
24.	Hyperthyroidism	H03BA02, H03BB01
25.	Hypothyroidism	H03AA01–H03AA02
26.	Irritable bowel syndrome	A07EC01–A07EC04, A07EA01–A07EA02, A07EA06, L04AA33
27.	Ischaemic heart disease: angina	C01DA02–C01DA14, C01DX16, C08EX02
28.	Ischaemic heart disease: hypertension	C07AA01–C07AA06, C07AA08–C07AB01, C07AB02—if PBS item code is not 8732N, 8733P, 8734Q, 8735R, C07AG01, C08CA01–C08DB01, C09DB01–C09DB04, C09DX01, C09BB02– C09BB10, C07AB03, C09DX03, C10BX03¶
29.	Incontinence	G04BD01–G04BD99
30.	Inflammation/pain	M01AB01–M01AH06
31.	Liver failure	A06AD11, A07AA11
32.	Malignancies	L01AA01–L01XX41
33.	Malnutrition	B05BA01–B05BA10
34.	Migraine	N02CA01–N02CX01
35.	Osteoporosis/Paget's	M05BA01–M05BB05, M05BX03, M05BX04, G03XC01, H05AA02
36.	Pain	N02AA01–N02AX02, N02AX06, N02AX52, N02BE51
37.	Pancreatic insufficiency	A09AA02
38.	Parkinson's disease	N04AA01–N04BX02
39.	Psoriasis	D05AA01–D05AA99, D05BB01, D05BB02, D05AX02, D05AC01–D05AC51, D05AX52
40.	Psychotic illness	N05AA01–N05AB02, N05AB06–N05AL07, N05AX07–N05AX13
41.	Pulmonary hypertension	C02KX01–C02KX05, PBS item code 9547L, 9605M
42.	Renal disease	B03XA01–B03XA03, A11CC01–A11CC04, V03AE02, V03AE03, V03AE05
43.	Smoking cessation	N07BA01–N07BA03, N06AX12
44.	Steroid-responsive disease	H02AB01–H02AB10
45.	Transplant	L04AA06, L04AA10, L04AA18, L04AD01, L04AD02
46.	Tuberculosis	J04AC01–J04AC51, J04AM01–J04AM99

*Benign prostatic hyperplasia medicines are tested for gender—must be male. Females suffering from bladder obstructions can be prescribed medicines used to treat benign prostatic hyperplasia. †Must have at least two medicines prescribed with one of those medicines having an ATC code from C03CA01–C03CC01 and the other having an ATC code from either C09AA01–C09AX99 or C09CA01–C09CX99. ‡Combination product for hyperlipidaemia and diabetes. §Can have medicine dispensed with an ATC code C03CA01–C03CC01 or C09AA01–C09A×99 or C09CA01–C09C×99, but not both, as this would indicate chronic heart failure. ¶Combination product for hyperlipidaemia and ischaemic heart disease: hypertension. N/A, not applicable.

Constructing a skin phenotype score

We derived a skin phenotype score by applying weights to individual phenotypic traits (skin color, propensity to skin burning and tanning, and natural hair color). The weights were derived from the coefficients of a binary logistic regression model in which the outcome was treatment for skin cancer, and the predictors were age, sex, and the phenotypic characteristics. We ascertained the outcome from the baseline questionnaire (self-reported history of skin cancer excision and/or cryotherapy), the MBS (evidence of treatment of keratinocyte cancer), and the QCR (evidence of melanoma diagnosis). The reference levels of the phenotypic characteristics were medium-colored skin that burns moderately and tans moderately, and light brown hair. The phenotypic score for each participant was computed as the sum of the coefficents (i.e., log odds ratios) for their phenotypic characteristics; refer to Table SC for the fitted values.

Table SC. Logistic regression model of the association between skin phenotypes and skin cancer outcome, adjusting for sex and age at baseline

Characteristic	N	log(OR)	95% CI	p-value
Skin color				
Medium	12,682	0	ref	
Fair	23,750	0.32	0.27, 0.38	<0.001
Olive/Dark	3,113	-0.56	-0.65, -0.47	<0.001
Black	26	-2.2	-3.6, -1.1	<0.001
Skin burning				
Burn moderately	13,352	0	ref	
Not burn	3,573	-0.76	-0.85, -0.67	<0.001
Burn a little	17,094	-0.30	-0.35, -0.24	<0.001
Burn badly	5,552	0.26	0.18, 0.35	<0.001
Skin tanning				
Tan moderately	19,531	0	ref	
Not tan	2,597	0.56	0.44, 0.69	<0.001
Tan a little	8,344	0.25	0.19, 0.32	<0.001
Tan deeply	9,099	-0.22	-0.28, -0.17	<0.001
Hair color				
Light brown	14,676	0	ref	
Red/Auburn	2,268	0.36	0.23, 0.49	<0.001
Dark brown	13,207	-0.10	-0.15, -0.04	<0.001
Blonde	5,558	0.01	-0.07, 0.08	0.9
Black	3,862	-0.54	-0.62, -0.46	<0.001

OR = Odds ratio, CI = Confidence interval

Supplementary Methods S2. Analyses of associations between participant characteristics and vitamin D testing, stratified according to time period (2011-2014 versus 2015-2019)

Follow-up and eligibility

The outcome for these analyses was the first vitamin D test after start of follow-up in each period. For the 2011–2014 period, we began follow-up one year after the date of consent. Follow-up ended at the earliest of: (1) the first vitamin D test; (2) the date of death; or (3) 31 December 2014. Participants were excluded if they had undergone a vitamin D test, and/or died in the 12 months prior to the start of the follow-up.

For the 2015–2019 period, follow-up started on 1 January 2015 and ended at the earliest of: (1) the first vitamin D test that occurred after 1 January 2015; (2) the date of death; or (3) 31 December 2019. Participants were excluded if they had undergone a vitamin D test in 2014 and/or died before 1 January 2015.

Explanatory variables

For these analyses, the following variables were of interest: gender, age, ancestry origin, skin phenotype, SEIFA, history of skin cancer treatment, ever been or newly prescribed with osteoporosis, antiepileptic medication, or menopausal hormone therapy (for women), number of GP visits 12 months before cohort entry, Rx-Risk comorbidity index at cohort entry.

The date of the first use of relevant medication and skin cancer treatment was compared against the date of cohort entry and the end of follow-up to derive the exposure status for each individual. Number of GP visits and Rx-Risk comorbidity index were calculated separately for the two periods based on the 12-month period before the start of follow-up.

Supplementary Methods S3. Clinical indications for vitamin D testing not available

The following clinical indications for vitamin D testing for adults in Australia could not be captured due to data unavailability in the Medicare Benefits Schedule database:

- increased alkaline phosphatase in otherwise normal liver function tests
- hyperparathyroidism, hypocalcaemia or hypercalcaemia, hypophosphataemia
- malabsorption (for example, cystic fibrosis, untreated coeliac disease, short bowel syndrome, or bariatric surgery)
- chronic renal failure or renal transplant recipients

Supplementary Results

Table S1. Average percent changes in age-standardized person-based rates of vitamin D testing in QSkin participants between 2011 and 2019; results presented for all participants and according to sex

Time segment	Average quarterly percent change (95% CI)
Person	
Q1/2011- Q1/2013	8.36 (6.02, 14.28)
Q1/2013- Q4/2014	-0.48 (-3.94, 3.46)
Q4/2014- Q3/2015	-13.69 (-16.47, 1.91)
Q3/2015- Q4/2019	3.03 (2.29, 3.8)
**Q1/2011- Q4/2019**	**1.95 (1.56, 2.56)**
Female	
Q1/2011- Q1/2013	7.67 (5, 16.38)
Q1/2013- Q4/2014	-0.58 (-5.03, 3.55)
Q4/2014- Q3/2015	-13.33 (-16.24, 3.41)
Q3/2015- Q4/2019	2.68 (1.7, 3.53)
**Q1/2011- Q4/2019**	**1.64 (1.18, 2.42)**
Male	
Q1/2011- Q1/2013	9.88 (5.97, 25.46)
Q1/2013- Q3/2014	0.22 (-12.14, 5.37)
Q3/2014- Q2/2015	-16.34 (-19.69, 5.92)
Q2/2015- Q4/2019	3.87 (2.2, 4.98)
**Q1/2011- Q4/2019**	**2.65 (1.95, 3.77)**

Average quarterly percent changes were estimated using Joinpoint regression.

Table S2. Sensitivity analysis: Distribution of most likely indication for the first vitamin D test, and result of the test according to whether test was indicated; including Rx-Risk comorbidity index ≥2 as an indication

Indication[1]	%[2]	Mean 25(OH)D (SD)[3]	n (%) deficient[3]
No apparent indication	32.2	69.8 (21.3)	631 (15.8)
Rx-Risk comorbidity index ≥2[4]	48.6	66.1 (22.7)	1,400 (23.0)
Low sun exposure (≤3.5 hour/week outdoors)	8.7	62.4 (21.4)	291 (27.2)
Obesity (body mass index ≥30 kg/m2)	8.0	62.3 (19.9)	250 (25.4)
Long-term glucocorticoid use[5]	1.7	63.4 (21.2)	53 (26.5)
Recently prescribed with osteoporosis/antiepileptic medication[6]	0.9	65.9 (23.2)	29 (25.9)

[1]Each participant was assigned only one indication for the vitamin D test. If a participant had two or more indications, the highest-ranked indication was assigned to that participant. The hierarchy in which we assigned the indication was osteoporosis/antiepileptic medication, long-term glucocorticoids use, Rx-Risk score ≥2, low sun exposure, and being obese.

[2]Include participants who were not missing any data required to determine whether the test was indicated (N=15,122).

[3]Include participants who were not missing the result of the first vitamin D test, or any data required to determine whether the test was indicated (total N=12,452, no clear indication, N=3,987, at least one indication, N=8,465).

[4]Derived from the 12 months before the first vitamin D test.

[5]Medication with Anatomical Therapeutic Chemical code H02AB dispensed at least 4 times in 12 months before the first vitamin D test.

[6]Prescribed within 90 days before the test.

Table S3. Associations between participant characteristics and having at least one vitamin D test, stratified by time period (prior to 2015 versus 2015 onwards)

Characteristic	2011-2014		2015-2019	
	N, N=35,250	aHR (95% CI)[1]	N, N=32,981	aHR (95% CI)[1]
Sex				
Male	16,880		16,066	
Female	18,370	2.74 (2.61, 2.88)	16,915	2.48 (2.38, 2.58)
Age at cohort entry (years)[2]				
<45	2,859	Ref	150	Ref
45 to <50	4,986	1.12 (1.01, 1.25)	4,372	0.98 (0.73, 1.31)
50 to <55	6,375	1.28 (1.16, 1.42)	5,208	1.07 (0.80, 1.43)
55 to <60	6,659	1.46 (1.33, 1.61)	6,103	1.18 (0.88, 1.57)
60 to<65	6,516	1.56 (1.41, 1.72)	6,469	1.29 (0.97, 1.73)
65+	7,855	1.79 (1.63, 1.97)	10,679	1.57 (1.18, 2.10)
Ancestry origin				
White European	32,882	Ref	30,813	Ref
Other[3]	2,054	1.27 (1.17, 1.38)	1,881	1.15 (1.07, 1.24)
Missing	*314*		*287*	
SEIFA category at baseline[4]				
1 - most disadvantaged	7,306	Ref	6,798	Ref
2	7,300	1.08 (1.01, 1.16)	6,791	1.10 (1.03, 1.16)
3	7,082	1.21 (1.13, 1.30)	6,568	1.18 (1.11, 1.26)
4	6,807	1.31 (1.23, 1.41)	6,435	1.25 (1.18, 1.33)
5 - least disadvantaged	6,755	1.60 (1.50, 1.71)	6,389	1.27 (1.20, 1.35)
Number of GP visits in the 12 months before cohort entry				
0	3,350	Ref	2,996	Ref
1	3,442	1.35 (1.19, 1.53)	2,812	1.42 (1.27, 1.58)
2+	28,458	1.67 (1.51, 1.85)	27,173	1.72 (1.57, 1.88)
Rx-Risk comorbidity index at cohort entry (unweighted)				
0	18,718	Ref	11,297	Ref
1	6,828	1.22 (1.15, 1.29)	7,103	1.18 (1.12, 1.25)
2+	9,704	1.48 (1.40, 1.57)	14,581	1.42 (1.35, 1.49)
Skin phenotype (predisposition to skin cancers)[5]				
Lowest risk	12,461	Ref	11,649	Ref
Medium risk	11,238	1.01 (0.95, 1.06)	10,541	0.94 (0.89, 0.98)
Highest risk	11,551	1.05 (1.00, 1.11)	10,791	1.00 (0.96, 1.05)
Skin cancer excision prior to baseline (self-report)				
None	21,066	Ref	19,817	Ref
1	4,879	1.08 (1.01, 1.15)	4,535	1.05 (0.99, 1.11)
2-10	7,504	1.12 (1.06, 1.18)	6,964	1.09 (1.04, 1.15)
>10	1,563	1.08 (0.97, 1.21)	1,453	1.06 (0.96, 1.17)
Missing	*238*		*212*	
Skin cancer cryotherapy prior to baseline (self-report)				
None	16,029	Ref	15,024	Ref
1-5	9,164	1.08 (1.02, 1.14)	8,575	1.08 (1.03, 1.13)
6-10	3,282	1.14 (1.05, 1.23)	3,031	1.11 (1.04, 1.19)
>10	6,604	1.12 (1.05, 1.19)	6,189	1.07 (1.01, 1.14)
Missing	*171*		*162*	

Characteristic	2011-2014		2015-2019	
	N, N=35,250	aHR (95% CI)[1]	N, N=32,981	aHR (95% CI)[1]
Diagnosed with in situ melanoma subsequent to baseline[6]				
No	34,862	Ref	31,951	Ref
Yes	388	1.00 (0.77, 1.29)	1,030	0.99 (0.87, 1.13)
Diagnosed with invasive melanoma subsequent to baseline[6]				
No	35,014	Ref	32,429	Ref
Yes	236	1.22 (0.91, 1.63)	552	1.16 (0.98, 1.37)
Treated for keratinocyte cancer subsequent to baseline[6]				
No	29,032	Ref	22,248	Ref
Yes	6,218	1.01 (0.95, 1.08)	10,733	1.05 (1.01, 1.10)
Ever been prescribed with osteoporosis medication[6]				
No	34,333	Ref	31,963	Ref
Yes	917	2.06 (1.86, 2.28)	1,018	1.92 (1.79, 2.07)
Recent osteoporosis medication[6,7]				
No	34,912	Ref	32,291	Ref
Yes	338	4.08 (2.99, 5.57)	690	5.83 (4.74, 7.17)
Ever been prescribed with antiepileptic medication[6]				
No	34,580	Ref	32,325	Ref
Yes	670	1.25 (1.07, 1.47)	656	1.50 (1.35, 1.66)
Recent antiepileptic medication[6,7]				
No	34,941	Ref	32,578	Ref
Yes	309	2.10 (1.24, 3.54)	403	2.63 (1.70, 4.08)
Ever been prescribed with menopausal hormone therapy[6,8]				
No	16,896	Ref	15,288	Ref
Yes	1,474	1.24 (1.12, 1.38)	1,627	1.25 (1.19, 1.32)
Recent menopausal hormone therapy[6,7,8]				
No	17,406	Ref	14,234	Ref
Yes	964	1.45 (1.09, 1.92)	2,681	1.45 (1.20, 1.76)

aHR = adjusted hazard ratio, CI = confidence interval, ref = reference category.

[1]All variables were adjusted for sex and age at cohort entry (with the exception of menopausal hormone therapy which was not adjusted for sex). Additional adjustments were as follow: SEIFA was adjusted for skin phenotype; BMI was adjusted for SEIFA; GP visits was adjusted for skin phenotype, SEIFA, and Rx-Risk index at cohort entry; menopausal hormone therapy was adjusted for skin phenotype and SEIFA; and other variables were adjusted for skin phenotype and SEIFA.

[2]Calculated separately for each cohort (2011-2014 and 2015-2019).

[3]Includes the following: Aboriginal and Torres Islander; Maori; South Sea Islander; Asian; African/Caribbean; and mixed.

[4]SEIFA (Socio-Economic Indexes for Area) score was produced for all QSkin participants based on their postcode at baseline, then categorized into quintiles.

[5]A skin phenotype score was calculated based on skin color, propensity to burn, propensity to tan, and natural hair color. We categorized the score into tertiles based upon the distribution of the score in QSkin participants.

[6]Prior to the first test.

[7]Events occurring within 90 days of first prescription considered.

[8]Restricted to women.

Table S4. Distribution of number of vitamin D tests and time between consecutive tests between 2011 and 2019, according to selected participant characteristics

Characteristics	N	n (%) with ≥1 tests (among all participants, N=35,250)	n (%) with ≥2 tests (among who had ≥1 test, N=9,099)	Participants with ≥2 tests	
				Median number of tests per person (25th, 75th percentile)	Median months between tests (25th, 75th percentile)
Sex					
Female	18,370	10,637 (57.9)	6,662 (62.6)	3 (2, 4)	11.9 (5.9, 22.1)
Male	16,880	5,108 (30.3)	2,437 (47.7)	3 (2, 4)	12.0 (5.8, 20.9)
Age at cohort entry (years)					
<45	2,859	1,100 (38.5)	580 (52.7)	3 (2, 4)	12.0 (6.1, 23.4)
45 to <50	4,986	2,022 (40.6)	1,092 (54.0)	3 (2, 4)	12.3 (6.0, 23.2)
50 to <55	6,375	2,697 (42.3)	1,512 (56.1)	3 (2, 4)	11.8 (5.9, 22.8)
55 to <60	6,659	2,971 (44.6)	1,740 (58.6)	3 (2, 4)	11.9 (5.9, 21.7)
60 to<65	6,516	3,010 (46.2)	1,755 (58.3)	3 (2, 4)	12.0 (6.0, 21.4)
65+	7,855	3,945 (50.2)	2,420 (61.3)	3 (2, 4)	11.8 (5.7, 20.7)
Ancestry origin					
White European	32,882	14,538 (44.2)	8,330 (57.3)	3 (2, 4)	12.0 (5.9, 21.9)
Other[1]	2,054	1,048 (51.0)	670 (63.9)	3 (2, 4)	11.7 (5.6, 20.9)
Missing	*314*				
Skin phenotype (predisposition to skin cancers)[2]					
Lowest risk	12,461	5,394 (43.3)	3,097 (57.4)	3 (2, 4)	11.9 (5.9, 21.7)
Medium risk	11,238	4,923 (43.8)	2,795 (56.8)	3 (2, 4)	11.9 (5.8, 21.3)
Highest risk	11,551	5,428 (47.0)	3,207 (59.1)	3 (2, 4)	12.0 (6.0, 22.3)
Skin cancer excision prior to baseline (self-report)					
No	21,066	9,226 (43.8)	5,267 (57.1)	3 (2, 4)	11.8 (5.8, 21.6)
Yes	13,946	6,401 (45.9)	3,761 (58.8)	3 (2, 4)	12.0 (6.0, 22.1)
Missing	*238*				
Skin cancer cryotherapy prior to baseline (self-report)					
No	16,029	6,871 (42.9)	3,880 (56.5)	3 (2, 4)	11.9 (5.8, 21.9)
Yes	19,050	8,785 (46.1)	5,167 (58.8)	3 (2, 4)	12.0 (5.9, 21.8)
Missing	*171*				

Characteristics	N	n (%) with ≥1 tests (among all participants, N=35,250)	n (%) with ≥2 tests (among who had ≥1 test, N=9,099)	Participants with ≥2 tests	
				Median number of tests per person (25th, 75th percentile)	Median months between tests (25th, 75th percentile)
Result of the first vitamin D test (nmol/L)					
<30	343	343 (100.0)	249 (72.6)	3 (2, 5)	6.7 (3.5, 13.6)
30 to <50	2,418	2,418 (100.0)	1,656 (68.5)	3 (2, 5)	9.7 (4.6, 18.1)
50 to <75	6,023	6,023 (100.0)	3,434 (57.0)	3 (2, 4)	12.1 (6.1, 22.5)
75+	4,168	4,168 (100.0)	2,224 (53.4)	3 (2, 4)	12.8 (6.9, 24.1)

[1]Includes the following: Aboriginal and Torres Islander; Maori; South Sea Islander; Asian; African/Caribbean; and mixed.

[2]A skin phenotype score was calculated based on skin color, propensity to burn, propensity to tan, and natural hair color. We categorized the score into tertiles based upon the distribution of the score in QSkin participants. People with skin phenotype 3 were the most vulnerable to skin cancers.

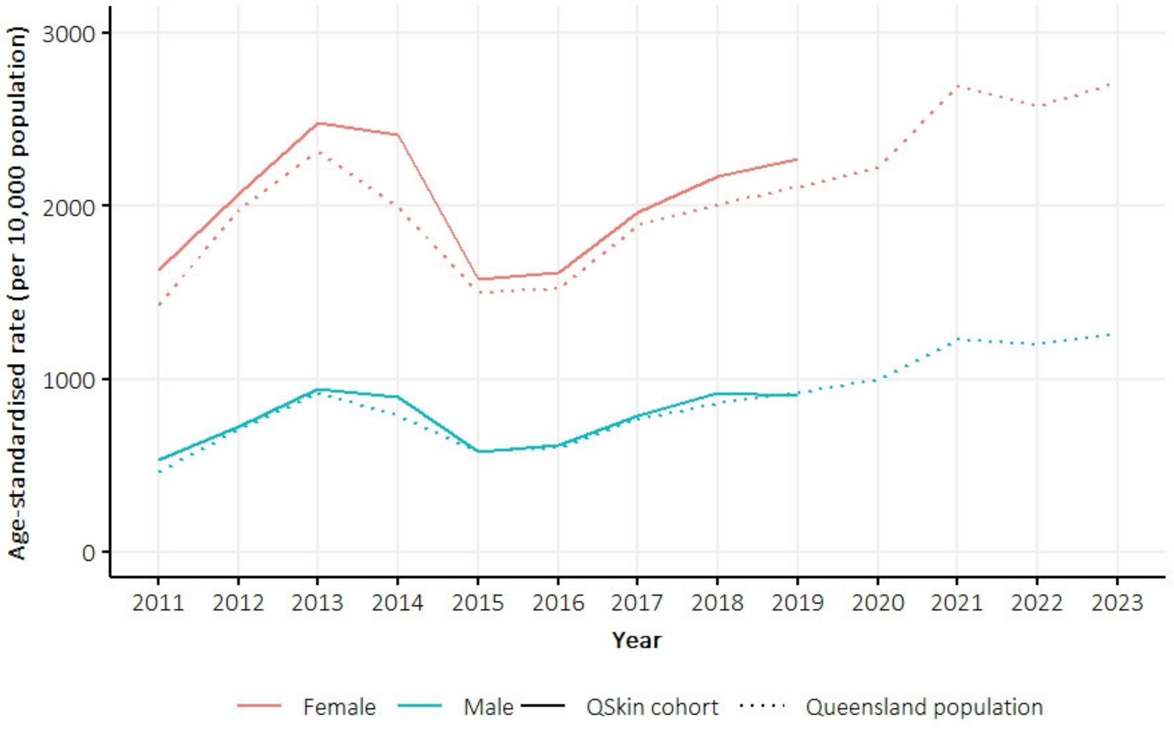

Figure S1. Test-based age-standardized rates[1] of vitamin D testing among the Queensland population[2] and the QSkin cohort[3] (aged 45-74)

[1]Standardized to Australian population 2001
[2]Data were retrieved from http://medicarestatistics.humanservices.gov.au/statistics/mbs_item.jsp
[3]Using MBS data only

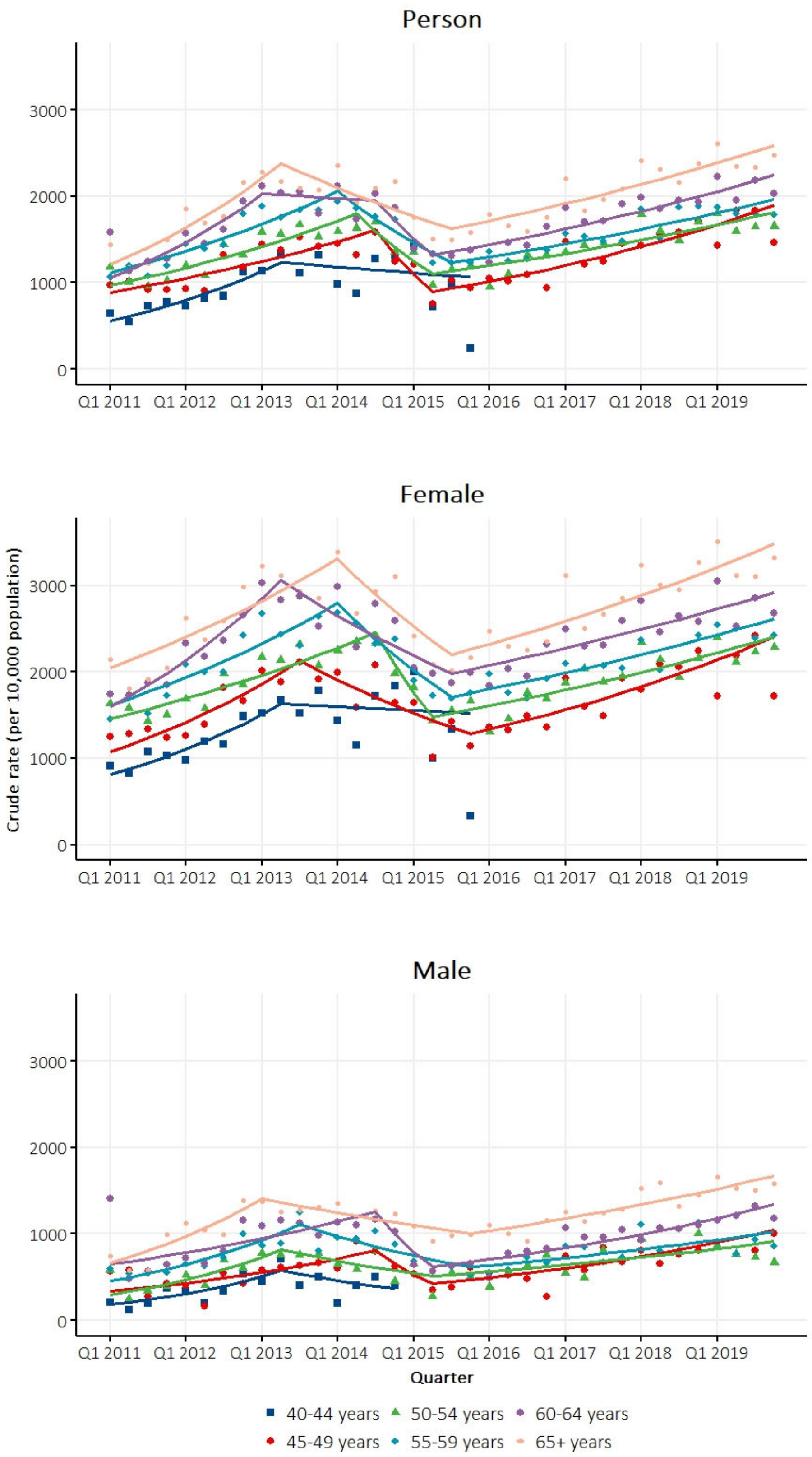

Figure S2. Age-specific person-based incidence rate of vitamin D testing between 2011 and 2019; data presented for all participants and according to gender

Note: Trend lines were estimated using Joinpoint regression models. The line for the 40-44 age-group (dark blue) is truncated at Q4/2014 because by Q1/2015 there were no longer any participants aged ≤44 years

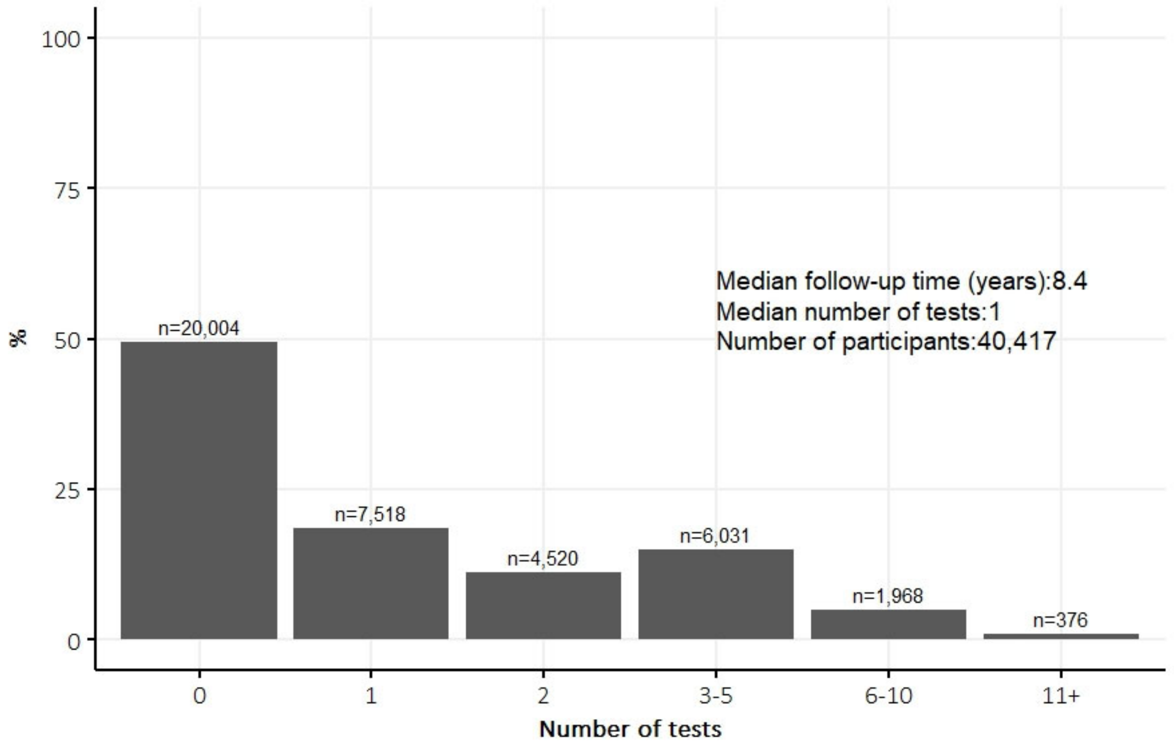

Figure S3. Number of tests per person between 2011 and 2019

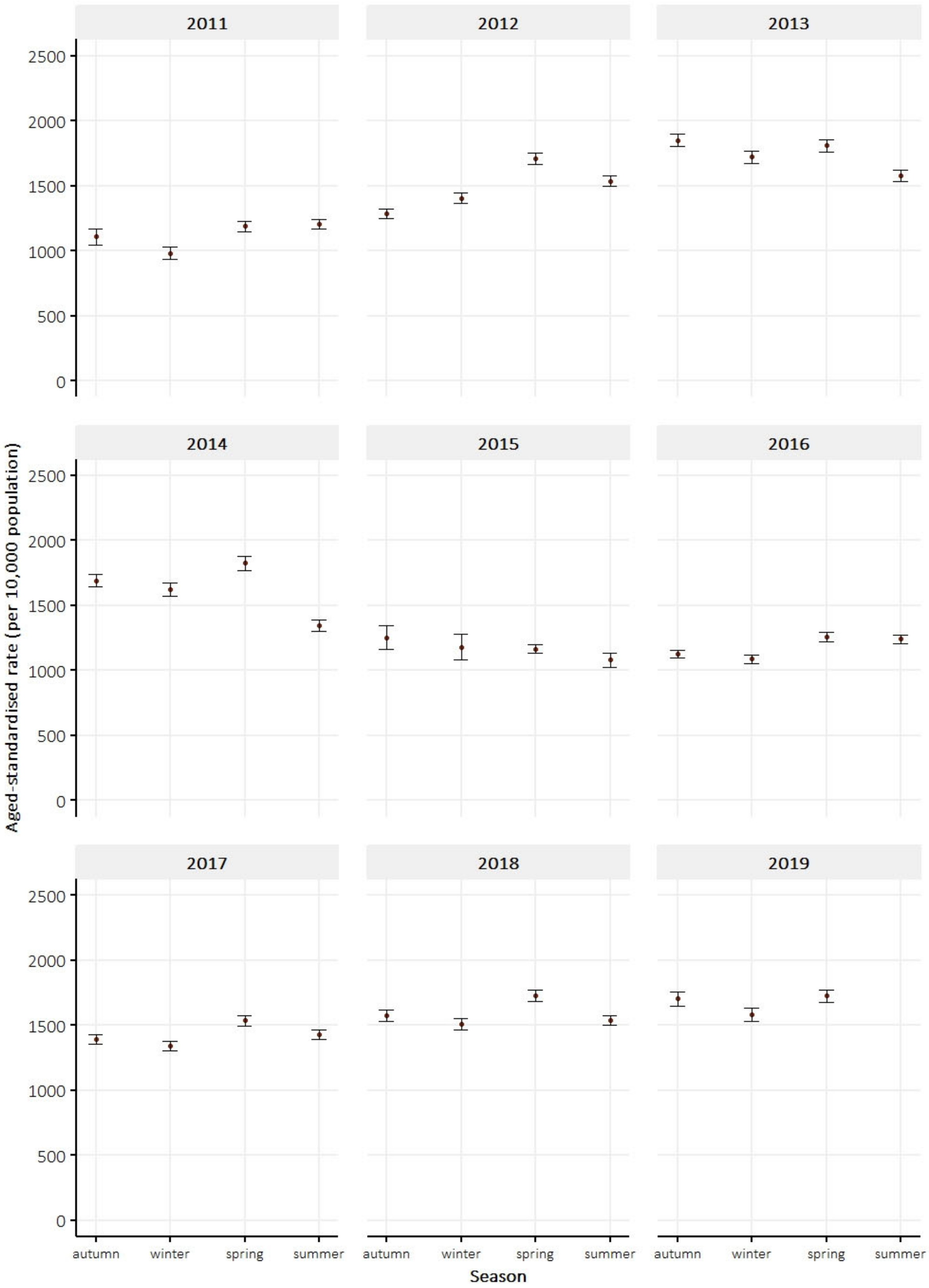

Figure S4. Person-based age-standardized incidence rate of vitamin D testing by season between 2011 and 2019. Error bars indicate ±1 standard error.

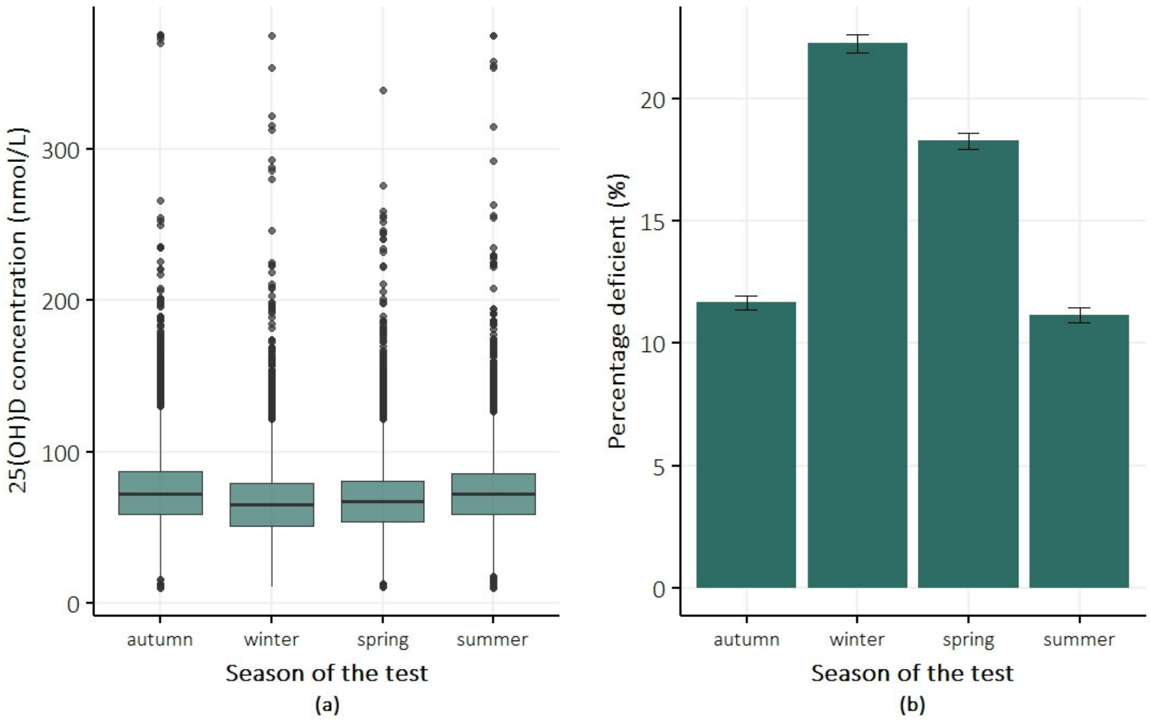

Figure S5. (a) 25(OH)D concentration; and (b) percentage of vitamin D tests indicating deficiency, according to season in tests conducted in QSkin participants between 2011 and 2019. Error bars indicate ±1 standard error.

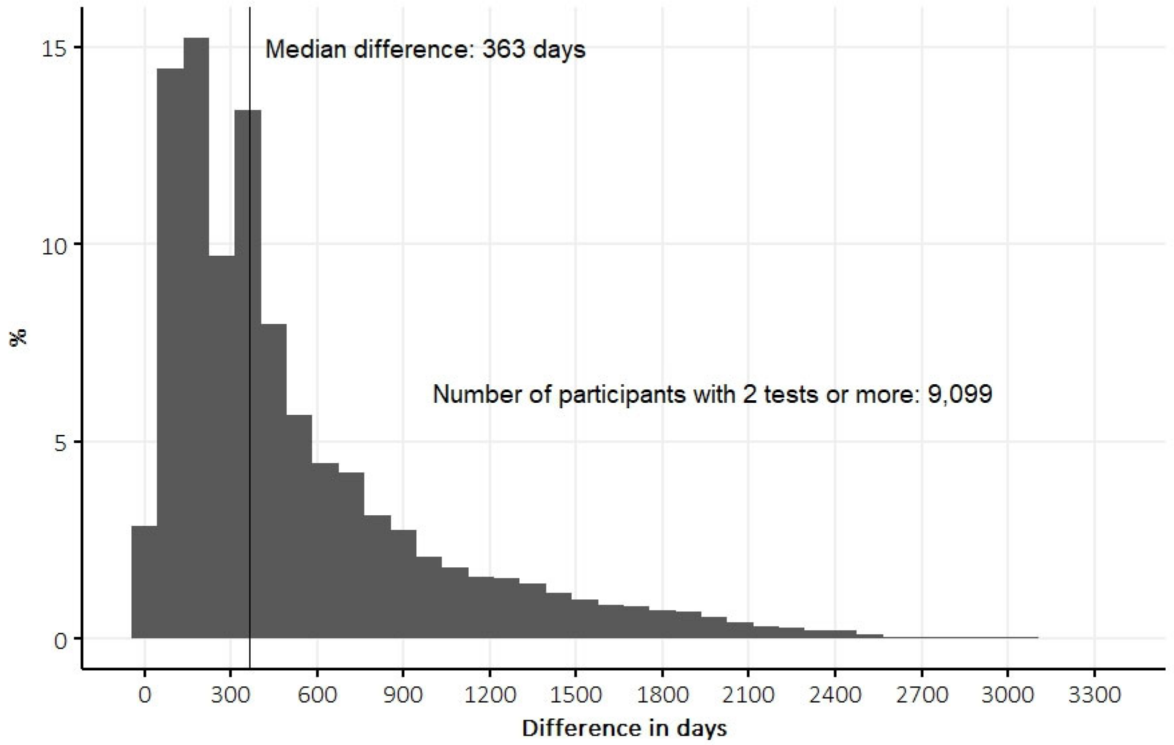

Figure S6. Time between consecutive vitamin D tests among participants who had at least 2 vitamin D tests between 2011 and 2019

Note: This figure excluded participants who had ≥1 test in the first year after the consent date

References

1. Pratt, N.L.; Kerr, M.; Barratt, J.D.; Kemp-Casey, A.; Ellett, L.M.; Ramsay, E.; Roughead, E.E. The validity of the Rx-Risk Comorbidity Index using medicines mapped to the Anatomical Therapeutic Chemical (ATC) Classification System. *BMJ Open* **2018**, *8*, e021122. https://doi.org/10.1136/bmjopen-2017-021122.
